# Elucidation of Gram-Positive Bacterial Iron(III) Reduction for Kaolinite Clay Refinement

**DOI:** 10.3390/molecules26113084

**Published:** 2021-05-21

**Authors:** Hao Jing, Zhao Liu, Seng How Kuan, Sylvia Chieng, Chun Loong Ho

**Affiliations:** 1 Department of Biomedical Engineering, Southern University of Science and Technology, Shenzhen 518055, China; 11930776@mail.sustech.edu.cn (H.J.); 11930810@mail.sustech.edu.cn (Z.L.); 2 Department of Mechanical and Material Engineering, Universiti Tunku Abdul Rahman, Selangor 43000, Malaysia; kuansh@utar.edu.my; 3 Department of Biological Sciences and Biotechnology, Universiti Kebangsaan Malaysia, Selangor 43000, Malaysia; sylvia@ukm.edu.my

**Keywords:** kaolin, iron(III)oxide, reduction, medium, secondary metabolites

## Abstract

Recently, microbial-based iron reduction has been considered as a viable alternative to typical chemical-based treatments. The iron reduction is an important process in kaolin refining, where iron-bearing impurities in kaolin clay affects the whiteness, refractory properties, and its commercial value. In recent years, Gram-negative bacteria has been in the center stage of iron reduction research, whereas little is known about the potential use of Gram-positive bacteria to refine kaolin clay. In this study, we investigated the ferric reducing capabilities of five microbes by manipulating the microbial growth conditions. Out of the five, we discovered that *Bacillus cereus* and *Staphylococcus aureus* outperformed the other microbes under nitrogen-rich media. Through the biochemical changes and the microbial behavior, we mapped the hypothetical pathway leading to the iron reduction cellular properties, and found that the iron reduction properties of these Gram-positive bacteria rely heavily on the media composition. The media composition results in increased basification of the media that is a prerequisite for the cellular reduction of ferric ions. Further, these changes impact the formation of biofilm, suggesting that the cellular interaction for the iron(III)oxide reduction is not solely reliant on the formation of biofilms. This article reveals the potential development of Gram-positive microbes in facilitating the microbial-based removal of metal contaminants from clays or ores. Further studies to elucidate the corresponding pathways would be crucial for the further development of the field.

## 1. Introduction

Kaolin is a clay mineral with stable chemical properties and good fire resistance, widely used in manufacturing paper, enamel, rubber, plastic, ceramics, coatings, cement, petrochemical, textile, and glass fiber [1,2]. However, kaolin often contains iron and aluminate contaminants, affecting its commercial value and product stability [2,3]. 

**Figure 1:**
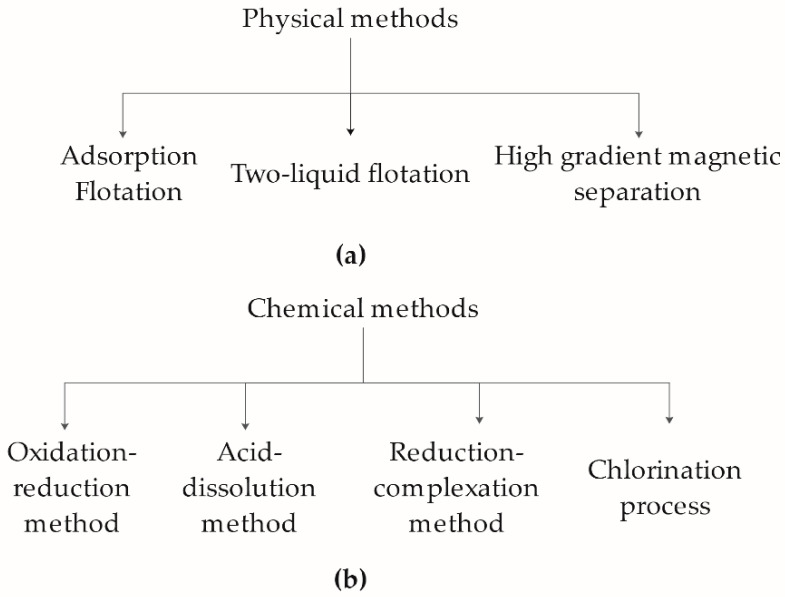
The traditional refining technology of kaolin. (**a**) Physical and (**b**) chemical methods used in isolating pure kaolin from iron contaminants.

The conventional kaolin refining technology can be divided into three approaches: physical (Figure 1a), chemical (Figure 1b), and microbial methods; but each approach presents many limitations. The physical process is ineffective in purifying heavily contaminated kaolin clay, relying on heavy equipment, trained personnel, and expansive energy consumption [3,4,5,6]. The more effective chemical method removes iron contaminants but employs various chemical reagents to facilitate the iron reduction process. This process is found to be hazardous to both personnel and the environment [7,8]. Lately, microbial-based treatment has become an emerging technology used for removing iron contaminants. This approach does not affect kaolin’s physical and chemical properties, and requiring low energy consumption with minimal impact on the environment. However, the processing time and the cultivation conditions (temperature, pH, medium conditions, and so forth) prevent the technology from moving forward.

Iron contaminants in kaolin exist mainly in the form of ferric(III)oxide, where an acid (<pH 3) is typically used in reducing ferric(III)oxide during kaolin purification. Ferrous ion, the reduced state of ferric ions, is soluble at a wide range of pH and remains stable at neutral or alkaline. In the presence of oxygen, ferrous ions rapidly oxidize to ferric ion, forming dense ferric(III)oxide solids. Many studies suggest that a microorganism with iron-reducing ability is attributed to its production of organic acids that dissolve ferric(III)oxide in the kaolin (reaction Equations (1) and (2)). For instance, co-culturing kaolin with *Aspergillus niger* for 40 h increased kaolin whiteness by over 50% [9]. However, this approach requires sustained acidification of the culture medium to pH 3. Iron-respiring bacteria (IRB) reduces ferric(III)oxide by electron transfer, where the resulting ferrous ions are used in microbial metabolism [10,11]. *Shewanella* is an example of microbial reduction of oxidized metals such as manganese and iron for microbial growth [12,13]. A variety of IRB have been isolated from sedimentary kaolin strata, providing a collection of potential microorganisms for the use of removing kaolin impurities, offering new and exciting avenues of microbial-based processes.

Fe_2_O_3_ + 6H^+^ → 2Fe^3+^ + 3H_2_O (1)

Fe^3+^ + e^−^ → Fe^2+^ (2)

This study investigates the ferric reduction properties of several Gram-positive and Gram-negative bacteria, aiming to understand the microbial bioleaching mechanism and the elicited changes in the surrounding microenvironment. Based on the biochemical analysis, we found two Gram-positive bacteria that showed promising iron reduction activity.

## 2. Results

We selected five bacteria to investigate the ferric(III)oxide reducing properties, comprising of two Gram-positive bacteria and three Gram-negative bacteria. Among them, *Bacillus cereus* UKMTAR-4 (*B. cereus*) and *Staphylococcus aureus* NCTC 6571 (*S. aureus*) are Gram-positive bacteria; whereas *Burkholderia thailandeensis* MSMB43 (*B. thailandeensis*), *Escherichia coli* K-12 (*E. coli*), and *Pseudomonas aeruginosa* DWW3 (*P. aeruginosa*) are Gram-negative. Ferric(III)oxide reducing properties of each microbe was observed throughout 14 days using both ferric(III)oxide and raw iron-contaminated kaolin clay from Jiangxi Province, China.

### 2.1. Microbial-Based Ferric(III)oxide-Reduction

**Figure 2:**
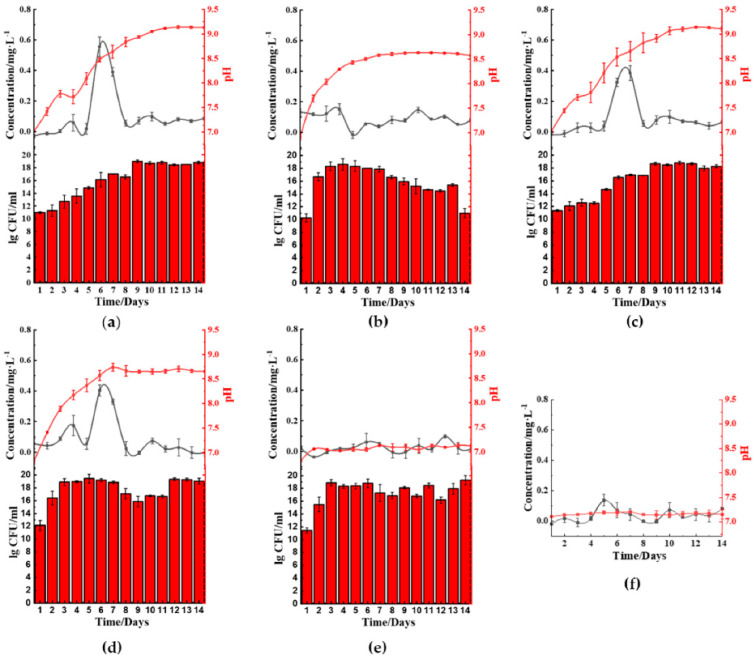
Microbial-mediated ferric(III)oxide reduction cultured in nitrogen-rich media. (**a**) *B. cereus*. (**b**) *S. aureus.* (**c**) *B. thailandeensis.* (**d**) *E. coli.* (**e**) *P. aeruginosa.* (**f**) Negative control (LB with ferric(III)oxide). (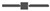): ferrous ion concentration; (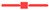): pH value, and bar chart: log_10_ colony forming unit (CFU)·mL^−1^; LB: lysogeny broth.

The five microbes cultured in ferric(III)oxide-lysogeny broth (LB) were monitored daily for their ferrous ion concentration, pH changes, and bacterial growth rate. Under a nitrogen-rich medium such as LB, most microbes raised the pH in the media by producing ammonia as a byproduct of nutrient utilization for growth and metabolism [14,15,16]. We discovered that *B. cereus* (Figure 2a), *B. thailandeensis* (Figure 2c), and *E. coli* (Figure 2d) showed elevated levels of ferrous ions on the sixth to eighth day before returning to basal levels. Correspondingly, the cultured medium’s pH values of these microbes exceeded pH 8.5 on day 6. Additionally, *B. cereus* and *B. thailandeensis* showed an observable lag phase and slower exponential growth during the first to sixth day. We similarly observed that the microbial cultures without ferric(III)oxide to a lesser extent also basified the media, below pH 8.5 (Figure S1). Additionally, there were no significant changes to the growth rate comparing the cultures of the microbes with and without ferric(III)oxide, indicating that there was no cellular toxicity caused by the ferric or ferrous ions produced (Figure S1). The iron reduction process successfully reduced 40.3%, 32.3%, and 33.3% of the total weight of ferric(III)oxide using *B. cereus*, *B. thailandeensis*, and *E. coli*, respectively. The loss of net ferric(III)oxide that is not reflected in the extracellular matrix suggests that the reduced ferrous ions were internalized for cellular metabolism.

*S. aureus* (Figure 2b) and *P. aeruginosa* (Figure 2e) showed no changes in the ferrous ion concentration, exhibiting unhampered growth. Interestingly, *S. aureus* cultures showed changes in pH where the cultures basified to approximately 8.5 before remaining stationary in that pH range.

### 2.2. Microbial-Based Reduction of Ferric(III)oxide Contaminants in Kaolin Clay 

**Figure 3:**
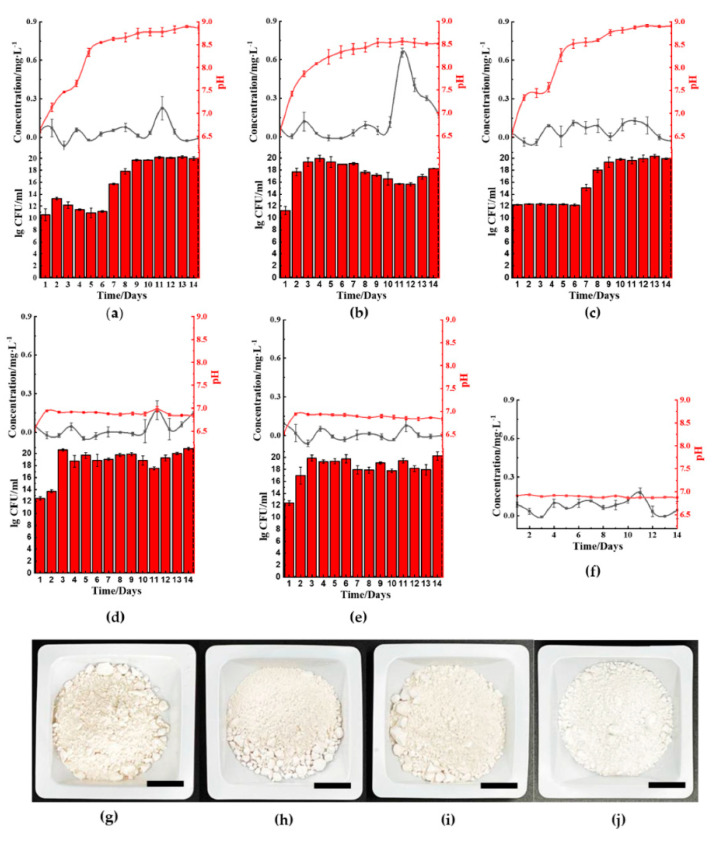
Microbial-mediated ferric(III)oxide reduction in kaolin clay cultured in nitrogen-rich media. (**a**) *B. cereus*. (**b**) *S. aureus.* (**c**) *B. thailandeensis.* (**d**) *E. coli.* (**e**) *P. aeruginosa.* (**f**) Negative control (LB with ferric(III)oxide). The retrieved kaolin clay after incubation including the (**g**) untreated, (**h**) *B. thailandeensis*, (**i**) *B. cereus*, and (**j**) *S. aureus* (Bar: 1 cm). (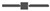): ferrous ion concentration; (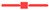): pH value, and bar chart: log_10_ CFU·mL^−1^; LB: lysogeny broth.

The ferric reduction properties of the five microbes cultured in kaolin clay-LB showed that *B. cereus* (Figure 3a) and *S. aureus* (Figure 3b) facilitated ferric(III)oxide reduction in kaolin clay on the 10th to 13th day of incubation. The surrounding medium’s pH increased to above pH 8.5 after day 6 and day 9, respectively. Cultures using *B. cereus* also showed a prominent lag phase until day 7 prior to seeing an increase of growth. Interestingly, cultures using *B. thailandeensis* (Figure 3c) showed similar pH and growth trends compared to the incubation with ferric(III)oxide. However, there was no noticeable increase in ferrous ions resulting in the *B. thailandeensis* cultures. The untreated kaolin clay was found to have 1.36% ferric(III)oxide contaminants. We found that using *S. aureus* removed 76.2% of the total ferric(III)oxide contaminants (Figure 3j). *B. cereus* and *B. thailandeensis* removed a total of 38.7% (Figure 3i) and 31.4% (Figure 3h) of ferric(III)oxide contaminants, respectively. The iron reducing process was visually observable from the kaolin clay retrieved from the treatment, showing improved whiteness from samples being treated with the three microbes when compared to the untreated kaolin clay (Figure 3g–i). 

There were no observable changes in the ferrous levels from cultures using *E. coli* (Figure 3d) or *P. aeruginosa* (Figure 3e). Similarly, there were no significant changes in both these cultures’ pH values while exhibiting unhampered growth. 

Based on the similarity, we are confident that the changes in the microbes’ pH conditions influence the response of the microbial bioleaching and impact the growth of the microbial cells. In both studies, *B. cereus* was a potential candidate to reduce ferric(III)oxide in kaolin clay, followed by *S. aureus.* Thus, to further understand the mechanism behind the bioleaching process, we conducted a closer inspection of *B. cereus* and *S. aureus* bioleaching properties under differing culturing conditions. We hypothesized that the different medium condition influences the microbial ability to leach these metal contaminants. 

### 2.3. Reduction Effect of B. cereus in Different Medium

Among the five microbes tested, *B. cereus* was the most efficient in reducing ferric(III)oxide. Thus, we investigated the corresponding pathways leading to the ferric(III)oxide reduction using different medium compositions.

**Figure 4:**
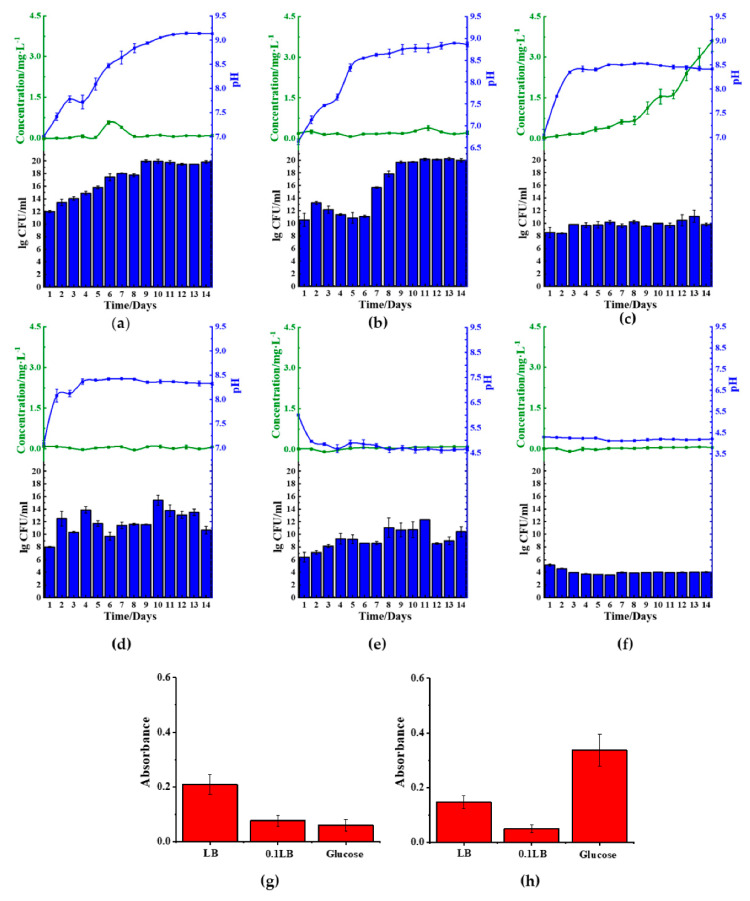
*B. cereus* ferric(III)oxide reduction and biofilm formation in different medium. Reduced ferrous, pH values, and growth rate of *B. cereus* cultured in (**a**) ferric(III)oxide-LB, (**b**) kaolin-LB, (**c**) ferric(III)oxide-0.1 LB, (**d**) kaolin-0.1 LB, (**e**) ferric(III)oxide-glucose, and (**f**) kaolin-glucose. The biofilm mass of *B. cereus* on (**g**) ferric(III)oxide and (**h**) kaolin clay. (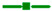): ferrous ion concentration; (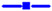): pH value, and bar chart: log_10_ CFU·mL^−1^; LB: lysogeny broth.

In order to achieve this objective, we cultured *B. cereus* in 1× LB (Figure 4a,b), 10-fold diluted LB (0.1 LB) (Figure 4c,d), and glucose medium (1.5% *w*/*v*). We found that *B. cereus* cultured with either LB or 0.1 LB showed increased basification of the medium, but lag phase disappeared in 0.1 LB (Figure 4, Figures S1 and S2). We further found that the use of 0.1 LB resulted in the best reduction of ferric(III)oxide salt, showing a total amassed value of 3.0 mg/L of ferrous ion in the medium. However, this particular trend was not observed in the kaolin introduced samples. We further found that *B. cereus* cultured in either ferric(III)oxide-0.1 LB or kaolin-0.1 LB was found to inhibit the microbial biofilm formation (Figure 4g,h) when compared to *B. cereus* biofilms formed on glass surfaces when grown in plain media (Figure S3). We noticed that in nitrogen-rich media, *B. cereus* readily interacted with the ferric(III)oxide and kaolin clay, where the cultures remained in suspension after remaining stationary for 10 min (Figure S4a). 

In contrast to the use of LB, glucose medium was found to acidify the medium, impacting the growth of *B. cereus* (Figure 4e,f). We found that the glucose rapidly decreased the pH to approximately pH 4 upon 24 h of culturing, resulting in poor microbial viability. Furthermore, ferric(III)oxide was found to negate the acidity’s effects on *B. cereus*, resulting in sustained growth, albeit lower than those cultured in LB, but did not restore the microbial cells’ ability to reduce the oxidize irons. 

### 2.4. Reduction Effect of S. aureus in Different Medium

**Figure 5:**
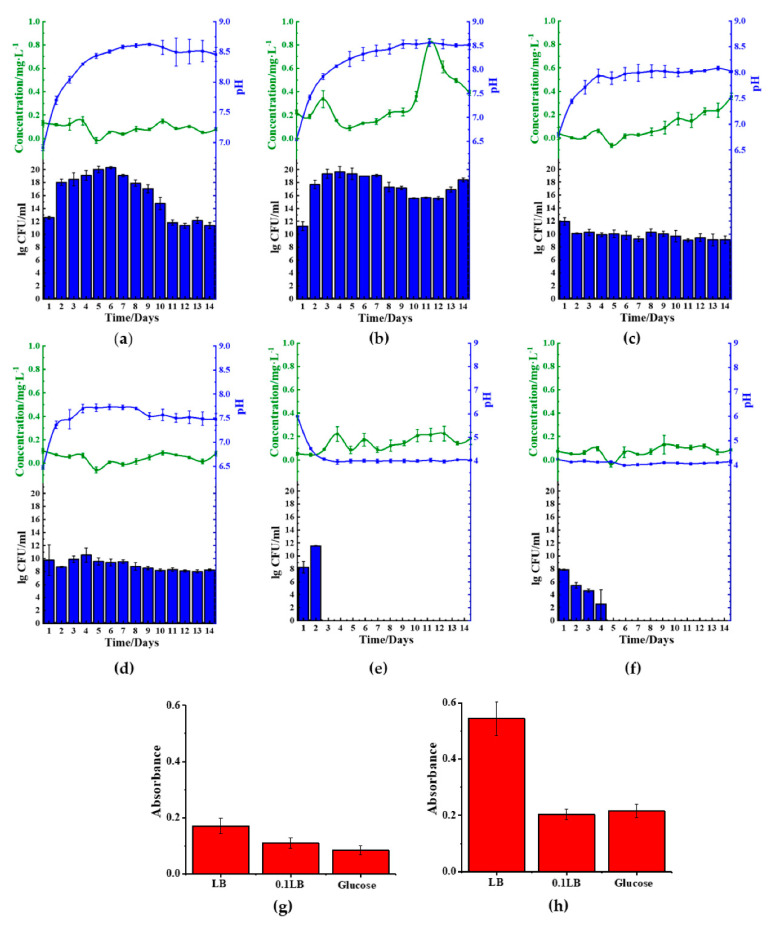
*S. aureus* ferric(III)oxide reduction and biofilm formation in different medium. Reduced ferrous, pH values, and growth rate of *S. aureus* cultured in (**a**) ferric(III)oxide-LB, (**b**) kaolin-LB, (**c**) ferric(III)oxide-0.1 LB, (**d**) kaolin-0.1 LB, (**e**) ferric(III)oxide-glucose, and (**f**) kaolin-glucose. The biofilm mass of *S.* *aureus* on (**g**) ferric(III)oxide and (**h**) kaolin clay. (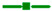): ferrous ion concentration; (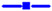): pH value, and bar chart: log_10_ CFU·mL^−1^; LB: lysogeny broth.

We also investigated the *S. aureus* behavior by regulating the different medium compositions. We found reduced ferrous was only present in kaolin samples cultured in LB medium (Figure 5a,b), indicating that the microbes could only reduce oxidized irons from these samples. Similar to the observation in *B. cereus*, the use of nitrogen-rich medium LB facilitated the medium’s basification. However, the 0.1 LB limited the growth of the microbial cells and, in doing so, reduced the rate of medium basification (Figure 5c,d). Similarly, the use of 0.1 LB inhibited cellular growth in *S. aureus*, showing lesser basification of the media (Figures S1 and S2). We similarly observed that the use of 0.1 LB also inhibited biofilm formation on the ferric(III)oxide and kaolin clay surfaces (Figure 5g,h), compared to the cultures on the glass surfaces (Figure S3). We also observed that in nitrogen-rich media, *S. aureus* also readily interacted with the ferric(III)oxide and kaolin clay, where the cultures remained in suspension after remaining stationary for 10 min (Figure S4b).

Similar to *B. cereus*, the use of the carbon-rich medium, 1.5% glucose (Figure 5e,f), resulted in the medium’s rapid acidification to approximately pH 4. The acidic environment hampered the growth of the microbes resulting in cellular death of *S. aureus.* Similarly, under such conditions, it was noticed that there was no significant reduction of ferric(III)oxide to ferrous ions. 

## 3. Discussion

We demonstrated that *B. cereus* is the most efficient in reducing iron contaminants in kaolin and ferric(III)oxide, followed by *S. aureus* which only strips iron contaminants from kaolin. In kaolin clay, crystalline ferric(III)oxide is found as small particles, while the amorphous ferric(III)oxide is presented on the surface of kaolin particles [17]. The ferric(III)oxide salt, on the other hand, exists in the α-ferric(III)oxide crystalline form. We are led to believe that *S. aureus* can only reduce amorphous ferric(III)oxide, whereas *B. cereus* can reduce crystalline and amorphous ferric(III)oxide. This selectiveness is due to the amorphous ferric(III)oxide’s hydrophilic nature, facilitating more straightforward iron reduction [18]. 

**Figure 6:**
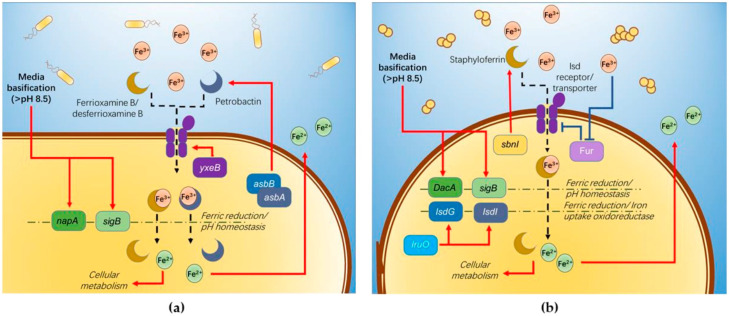
Hypothesized pathway involved in bacterial reduction of ferric ions. (**a**) *B. cereus*. (**b**) *S. aureus*.

Our findings on *B. cereus-*mediated ferric(III)oxide reduction in differing mediums reveal that a nitrogen-rich medium increases the iron reduction in both the ferric(III)oxide salt and kaolin. It is fascinating that the 0.1 LB showed an improved accumulation of ferrous ions in the medium. In the presence of ferric(III)oxide salt or kaolin clay, *B. cereus* triggers further basification of the media, causing the microbes to readily adhere to the crystalline ferric(III)oxide surface [19] (Figure 6a). In response, *yxeB*(an iron(II+) hydroxamate-binding protein) shuttles the ferric oxide into the microbial cytoplasm through the means of siderophores ferrioxamine B/desferrioxamine B [20]. Additionally, *B. cereus* regulates iron metabolism using the *asb* gene cluster producing the siderophore petrobactin used in combating oxidative stress, iron acquisition, and virulence [21,22,23]. The internalized ferric ions are then hypothesized to be reduced to the ferrous state through reduction caused by pH changes and superoxide dismutase. The medium’s basification results in the upregulation of stress response factor, *sigB* and Na^+/^H^+^ antiporter *napA* [24]. These two genes are involved in ablating the increased alkaline environment around the *B. cereus* involved in pH homeostasis in the alkaline pH range [25]. To facilitate this process, iron(III)oxide is reduced to the ferrous ion state, contributing protons for neutralizing the alkaline environment. Additionally, the reduction of ferric to ferrous ions via superoxide dismutase A facilitates iron uptake crucial to the growth and sporulation of *B. cereus* spores. Our observation on the *B. cereus* growth indicates that the reduced ferrous ions are depleted rapidly during microbial expansion, where the microbe’s upper growth limit inhibits further reduction and uptake of iron ions. Similarly, the use of 0.1 LB restricts the rapid expansion of microbial growth, resulting in the observed increased extracellular build-up of ferrous in the medium. Further contributed by the medium’s alkaline state, the ferrous ions remain in their reduced state in the extracellular matrix.

*S. aureus-*mediated ferric(III)oxide reduction is only observed in a nitrogen-rich medium. However, in the study, culturing *S. aureus* in 0.1 LB resulted in a cellular deficit. This phenomenon can be attributed to shifting the microbial lifestyle to form biofilms due to the limiting nutrient. *S. aureus* employs a similar approach in iron(III)oxide metabolism (Figure 6b). Previous studies found that *S. aureus* readily adheres to amorphous ferric ions such as haem, hemoglobin, and lactoferrin [26], triggering the upregulation of iron-regulated surface determinant (Isd) proteins via the Fur repressor inactivation [27]. The Isd system binds ferric ions through surface receptor interactions and internalizes using lipoproteins and membrane permease. The Isd-internalized ferric ions are bound to staphyloferrin, a siderophore produced by the *sbn* gene cluster. *SbnI* is a DNA-binding regulatory protein that senses iron to regulate the synthesis of staphyloferrin [28,29]. The ferric-bound stapyloferrin is reduced to ferrous through interactions with iron uptake oxidoreductase (IruO), a nicotinamide adenine dinucleotide phosphate (NADPH)-dependent reductase. IruO functions as an electron donor to degrading enzymes IsdG or IsdI, releasing ferric ions and reducing them to ferrous ions [30]. Additionally, the surrounding medium’s basification triggers the upregulation of *sigB* and *DacA*, a deadenylate cyclase enzyme used in regulating nitrogen metabolism and promoting ammonium and glutamine uptake [31,32,33]. The increased cellular base then reduces ferric ions to ferrous to balance the internal cellular pH [34]. The resulting ferrous ions used in the *S. aureus* cellular metabolism result in the loss of detectable ferrous ions in the extracellular matrix.

The study of these Gram-positive microbes revealed that the iron-reducing mechanism differs from other previously reported Gram-negative microbes. Previous studies on Gram-negative bacteria such as *Pseudomonas* and *Shewanella oneidensis* showed that the reduction of iron metals relies on producing organic acids, resulting in the acidification of the surrounding medium. These studies often use sugar (e.g., glucose, lactose, maltose, starch, sucrose, or molasses) as an energy source, increasing acidification. 

Based on these findings, we believe that the Gram-positive microbes facilitate the initial binding of microbes on the clay by altering the metal clays’ surface charges through the medium’s basification. Particularly in reducing crystalline ferric(III)oxide, the basification process’s role increases the salt’s surface charges to facilitate microbial colonization on the surface of the metal ores needed for electron transfer. Although both microbes are Gram-positive, the oxide iron reduction method differs slightly between the two microbes, evidenced by the two microbes’ differing preferences to strip crystalline and amorphous ferric(III)oxide.

## 4. Materials and Methods

### 4.1. Materials and Strains

All culturing media ingredients were purchased from Oxoid (Basingstoke, Hampshire, United Kingdom), Macklin (Shanghai, China) and Aladdin (Shanghai, China). Biochemical assay solutions were purchased from Aladdin (Shanghai, China), Dieckmann (Shenzhen, Guangdong, China) and Shanghai lingfeng chemical reagent Co., LTD (Shanghai, China). Raw kaolin was acquired from the local Chinese mines in Jiangxi Province, China.

Microbial strains *Pseudomonas aeruginosa* DWW 3 and *Escherichia coli* K-12 were acquired as a gift from Yang Liang, SUSTech; *Burkholderia thailandeensis* MSMB43 and *Staphylococcus aureus* NCTC 6571(from ATCC 9144) were acquired from the in-house microbial bank; while *Bacillus cereus* UKMTAR-4 was acquired as a gift from Sylvia Chieng, Universiti Kebangsaan, Malaysia.

The prepared medium included lysogeny broth, LB (10 g tryptone, 5 g yeast extract, and 10 g sodium chloride per liter); 10× diluted lysogeny broth, 0.1 LB (1 g tryptone, 0.5 g yeast extract, and 1 g sodium chloride per liter); and glucose medium (1.5% glucose *w*/*v*). Cultures were prepared using double distilled water and sterilized at 121 °C, 20 min prior to use. All microbial seed cultures were prepared in LB medium, incubated at 37 °C overnight. 

### 4.2. Equipments

Qianyan shaker (Changsha, Hunan, China), Yiheng constant temperature incubator (Shanghai, China), Yuanxi ultraviolet spectrophotometer (Shanghai, China), Eppendorf centrifuge (Hamburg, Germany), Tecan microplate reader (Männedorf, Canton of Zurich, Switzerland), Mettler pH meter (Zurich, Canton of Zurich, Switzerland).

### 4.3. Microbial Culturing Conditions

Microbes were cultured in lysogeny broth, LB, 0.1 LB medium, and glucose medium against ferric(III)oxide or kaolin ore. Briefly, 50 mL of culture medium was added with 0.3 g of ferric(III)oxide or 1g of kaolin powder and inoculated with the respective microbes to a final optical density of 0.02 (λ = 600 nm). Triplicates of each cultures were incubated under continuous shaking at room temperature and 160 rpm. Time-course sampling was conducted throughout the 14 days of incubation at 24 h intervals, sampling a total of 4% of the total culture volume. Sampled cultures were subjected to various biochemical tests as elaborated below. Negative control was prepared using sterile cultures subjected to the same conditions.

### 4.4. Ferrous Ion and pH Measurement

Daily samples were flash frozen for end point measurement. The ferrous ion concentration was quantified using 1,10-phenanthrolinet (reaction Equation (3)).

Fe^2+^ + 3(phen) = [Fe(phen)_3_]^2+^ (3)

Samples were tested using a mixture of the sample to 1,10-phenanthroline, ammonium acetate, and sodium fluoride ratio of 1:2:2:5. The mixture was left to incubate in the dark under room temperature for 1 h prior to measurement at absorption wavelength of 492 nm. The ferrous ions formed a stable [Fe(phen)_3_]^2+^ complex that was orange in color.

The experimental data of ferrous ion concentration presented in the content have subtracted the value of ferrous ion concentration in the medium at the corresponding time.

The pH was measured directly by a pH meter.

### 4.5. Bacterial Growth Rate Measurement

Daily microbial samples were subjected to serial dilution conducted in an aseptic environment. Serial dilutions were conducted from 1 to 20 orders of magnitude and spread on LB agar without antibiotics. The agar were incubated at 37 °C overnight. The number of colony forming units (CFU) were recorded along with the dilution factor. 

### 4.6. Determination of Total Ferric(III)oxide Content in Kaolin Clay

The kaolin clay was treated with 37% (*w*/*v*) hydrochloric acid for 12 h at room temperature to facilitate ferric(III)oxide reduction within the clay. The resulting solution was added to hydroxylamine hydrochloride at a 1:1 ratio to reduce ferric ions (reaction Equation (4)). Ferrous ions concentration was determined using 1,10-phenanthroline.

2Fe^3+^ + 2NH_2_OH·HCl = 2Fe^2+^ + N_2_ ↑ + 2H_2_O + 4H^+^ + 2Cl^−^ (4)

### 4.7. Determination of Total Reduced Ferric(III)oxide

Ferric(III)oxide or kaolin clay from 14 day cultures were retrieved by centrifugation and washed with ethanol to remove attached microbes. The precipitates were then incubated at 60 °C for 48 to 60 h, until there were no observable changes in weight. The final weight of the retrieved precipitates were compared to the initial weight of ferric(III)oxide/kaolin clay added to the cultures to determine the amount of reduced iron.

### 4.8. Determination of Microbial Biofilm Formation

Microbial biofilms were stained using crystal violet solution. Briefly, glass surfaces, kaolin clay, or ferric(III)oxide with microbial biofilm growth were stained with crystal violet solution (1 g/L) for 30 min, followed by vigorous washing with distilled water twice. The stained surface was left to dry at 60 °C for 10 h, before being resuspended in 10 mL of absolute ethanol. The resulting solution was measured at an absorption wavelength of 595 nm. 

## 5. Conclusions

The current report presents an initial investigation on bioleaching properties of five different microbial abilities to reduce ferric(III)oxide to ferrous ions. This work aims to identify the conditions governing the ferric(III)oxide reducing properties and use the culturing conditions to map out the potential pathways involved in the ferric(III)oxide reduction. We discovered that Gram-positive microbes *B. cereus* and *S. aureus* showed improved reduction compared to the other three microbes. We found that the use of varying culturing conditions impacted the iron-reducing capabilities of the microbes. Comparatively, the use of nitrogen-rich medium elicited ferric reduction by these Gram-positive microbes. Incubated kaolin with *B. cereus* in nitrogen-rich medium resulted in close to 80% removal of the oxidized iron contaminants, whereas *S. aureus* removed approximately 30%. The use of these Gram-positive microbes presented limited risk to its users and the environment as the treated kaolin were calcined at high temperatures, rendering these microbes inactive. At present, more studies on the metabolic pathways need to be investigated to fully understand the conditions needed to use these microbes for microbial-based kaolin treatment. It is possible to harness these microbes to efficiently remove the contaminating oxidized iron from kaolin clay using an environmentally friendly manner through a better understanding of the pathway.

## Data Availability

Supplementary Information is available for this paper at the corresponding webpage.

## Supplementary Materials

The following are available online, Figure S1: Changes in pH values and growth rates of microbial cultures in LB medium, Figure S2: Changes in pH values and growth rates of *B. cereus* and *S. aureus* cultures in 0.1 LB and glucose medium, Figure S3: Biofilm growth in the control group on a glass surface under different culturing conditions, Figure S4: Biofilm formation and culture appearance of *B. cereus* and *S. aureus* in different medium.

## Sample Availability

*Bacillus cereus* UKMTAR-4 is available from Sylvia Chieng’s research group. All other samples and strains are available from the Ho Chun Loong’s research group.
