# Supplementary material for: Elucidation of Gram-Positive Bacterial Iron(III) Reduction for Kaolinite Clay Refinement
Source: Molecules. 2021 May 21;26(11):3084. doi: 10.3390/molecules26113084 (PMC8196777; doi:10.3390/molecules26113084)
Supplement: Supplementary file 1 [file molecules-26-03084-s001.zip › molecules-1205532-supplementary.pdf]

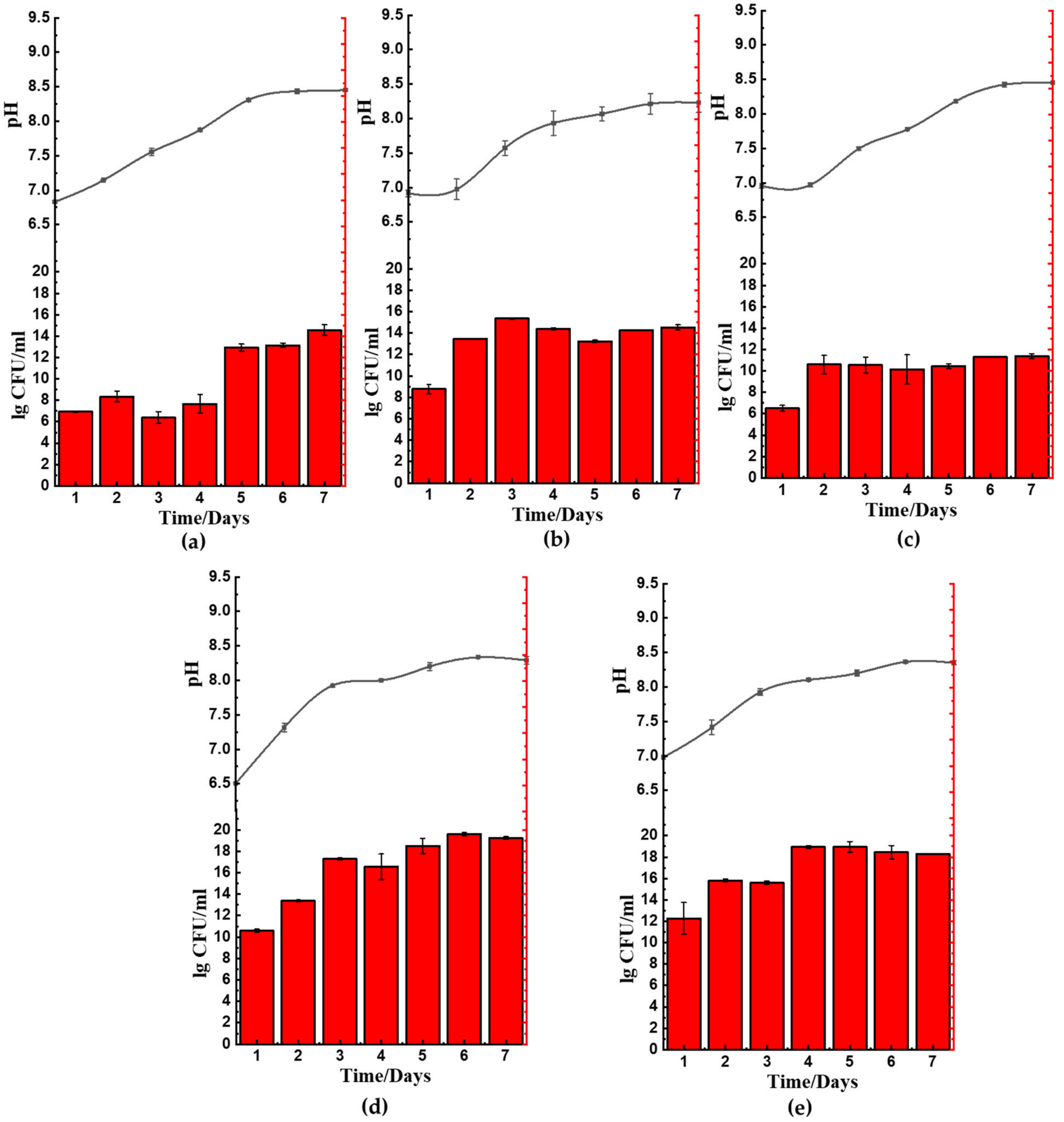

Figure S1. pH values and growth rates of five bacteria in LB medium. (**a**) *B. cereus*. (**b**) *S. aureus*. (**c**) *B. thailandeensis*. (**d**) *E. coli*. (**e**) *P. aeruginosa*. [(—■—) : pH value; bar chart: $\log_{10}$ CFU·mL^{-1}.

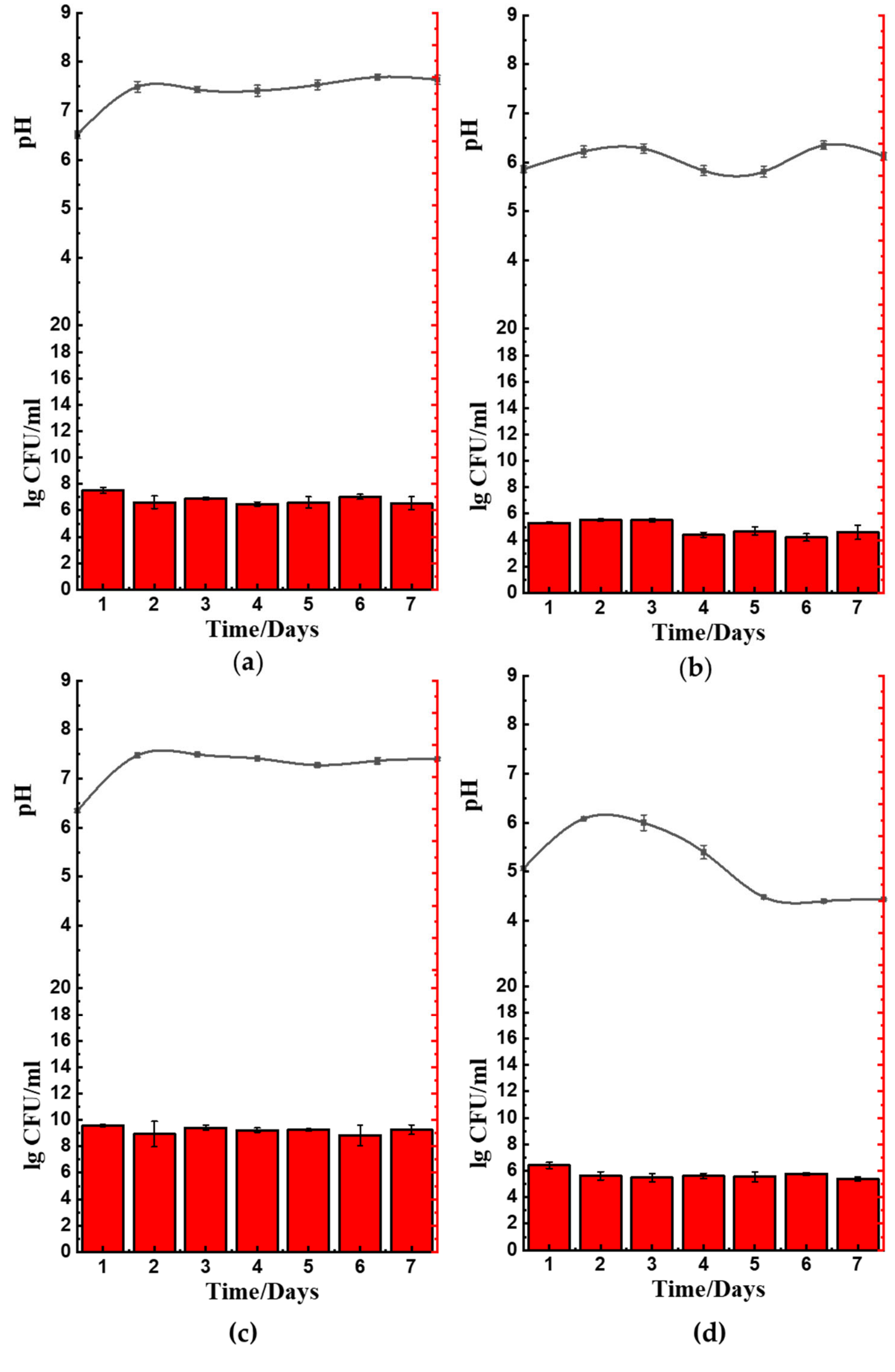

Figure S2. pH values and growth rates of *B. cereus* and *S. aureus* in different medium. (**a**) *B. cereus* in 0.1 LB. (**b**) *B. cereus* in glucose. (**c**) *S. aureus* in 0.1LB. (**d**) *S. aureus* in glucose. [(—■—) : pH value; bar chart: $\log_{10}$ CFU·mL^{-1}.

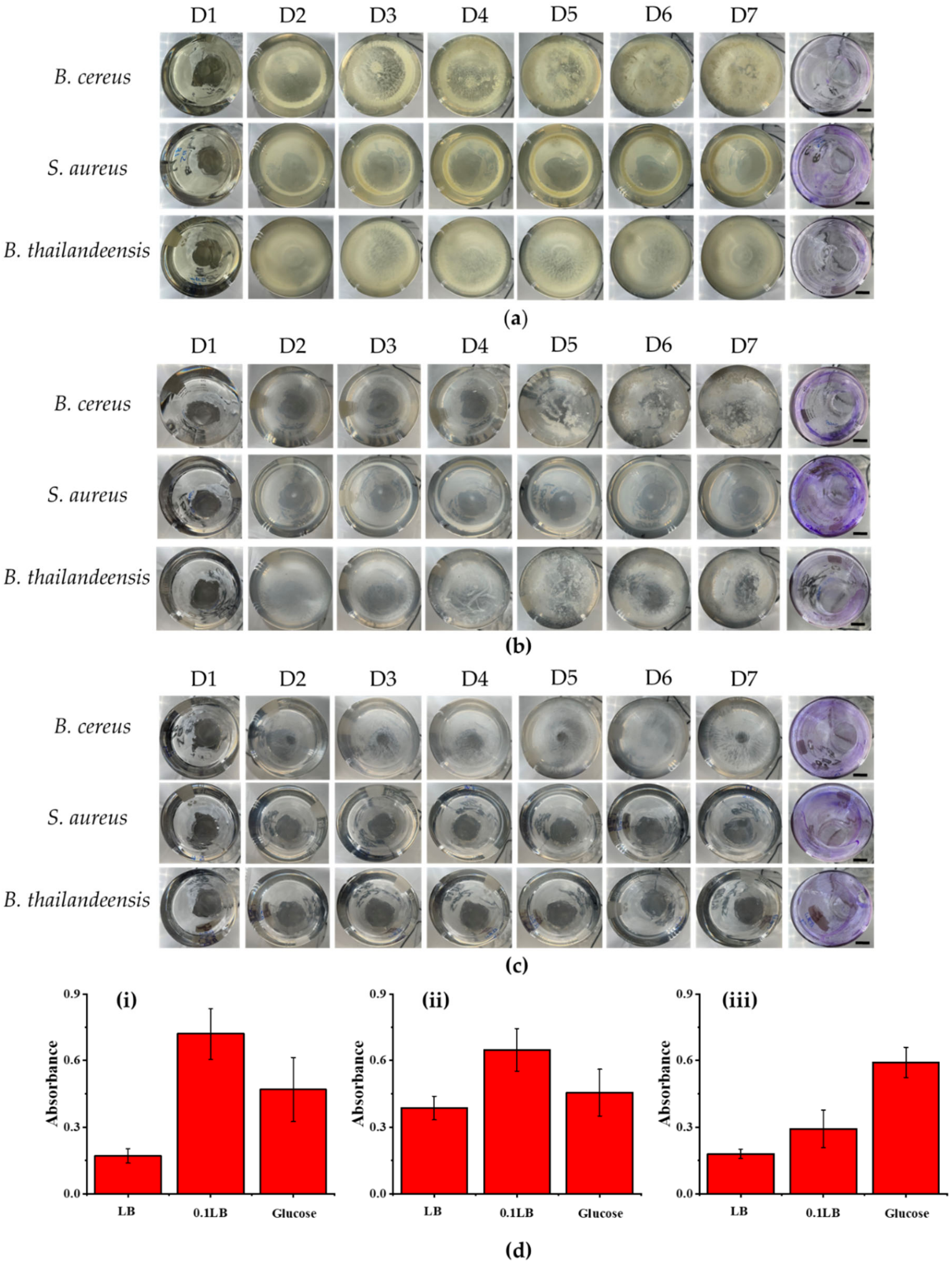

Figure S3. Biofilm growth in the control group without ferric(III)oxide and kaolin. (**a**) LB. (**b**) 0.1LB. (**c**) glucose. (**d**) The absorbance of the solution after dissolving the crystal violet stained biofilm. “D1 to D7” represented the first day to the seventh day of the experiment. The figures after the D7 of (**a**), (**b**) and (**c**) were stained biofilm with crystal violet. Bar: 1cm. The growth of the biofilm can be seen in the figure. [(**i**) *B. cereus*, (**ii**) *S. aureus*, (**iii**) *B. thailandeensis*.].

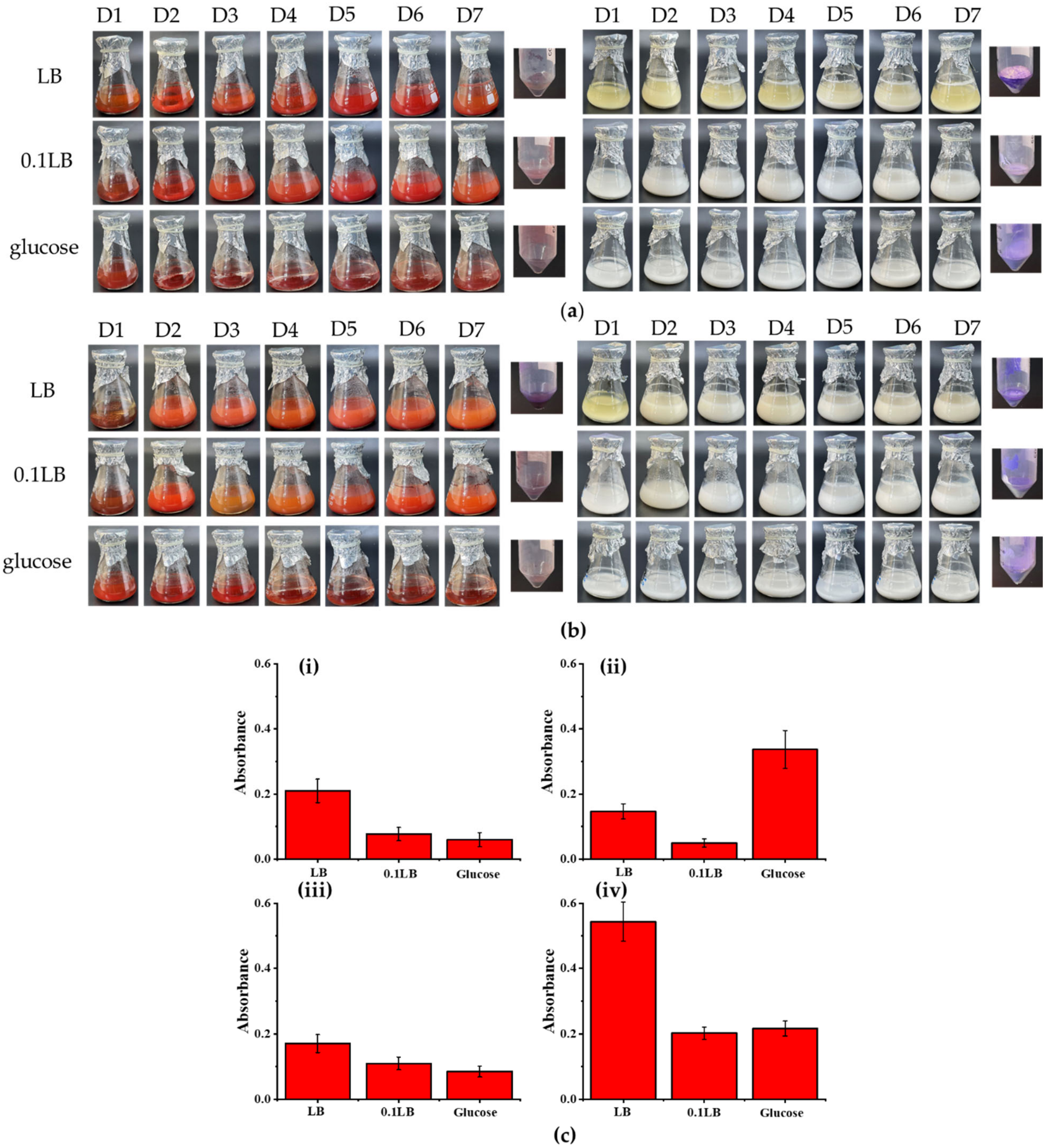

Figure S4. *B. cereus* and *S. aureus* reduced ferric(III)oxide and kaolin in different medium. (**a**) *B. cereus*. (**b**) *S. aureus*. (**c**) The absorbance of the solution after dissolving the crystal violet stained biofilm on the precipitate. "D1 to D7" represented the first day to the seventh day of the experiment. The figures of tube after D7 were the results of crystal violet staining for the precipitate after culture. The solution becomes uniform during the bacterial leaching process. In the figure, left is ferric(III)oxide and right is kaolin. [(i) ferric(III)oxide reduced by *B. cereus*, (ii) kaolin reduced by *B. cereus*, (iii) ferric(III)oxide reduced by *S. aureus*, (iv) kaolin reduced by *S. aureus*.].
